# Mind the Pitfall: Solitary Nodular Fasciitis Mimicking Extra-Nodal Manifestation of Hodgkin Lymphoma on [^18^F]FDG PET/CT

**DOI:** 10.3390/diagnostics14080783

**Published:** 2024-04-09

**Authors:** Suginthan Markandu, Arne Blickle, Caroline Burgard, Marc Remke, Katrin Altmeyer, Mathias Wagner, Samer Ezziddin, Florian Rosar

**Affiliations:** 1 Department of Nuclear Medicine, Saarland University, 66421 Homburg, Germany; arne.blickle@uni-saarland.de (A.B.); caroline.burgard@uks.eu (C.B.); samer.ezziddin@uks.eu (S.E.); florian.rosar@uks.eu (F.R.); 2 Department of Pediatric Oncology and Hematology, Saarland University, 66421 Homburg, Germany; marc.remke@uks.eu; 3 Department of Radiology, Saarland University, 66421 Homburg, Germany; katrin.altmeyer@uks.eu; 4 Department of Pathology, Saarland University, 66421 Homburg, Germany; mathias.wagner@uks.eu

**Keywords:** nodular fasciitis, Hodgkin lymphoma, FDG PET/CT, pitfall

## Abstract

We report a [^18^F]fluorodeoxyglucose positron emission tomography/computed tomography ([^18^F]FDG PET/CT) scan of a 17-year-old male presenting increased focal glucose metabolism of a histologically proven solitary nodular fasciitis mimicking an extranodal manifestation of Hodgkin lymphoma. This interesting image should draw attention to considering nodular fasciitis as a possible pitfall in the staging of malignant diseases.

**Figure 1:**
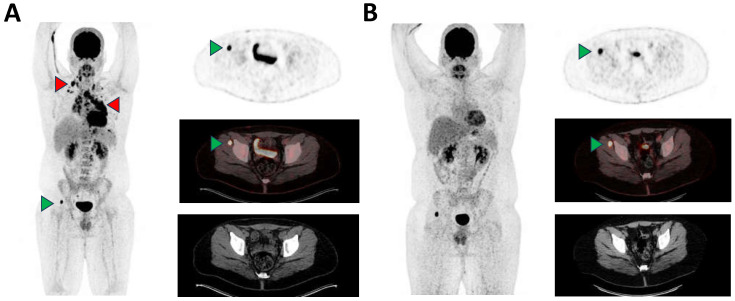
A 17-year-old male with histologically confirmed EBV-negative nodular sclerosing Hodgkin lymphoma (NSHL) was presented to our nuclear medicine department for staging. The [^18^F]FDG PET/CT showed extensive cervical, thoracic (mediastinal, hilar, pericardial, axillary and retrocrural) and abdominal (retroperitoneal, left iliac) nodal and extranodal pleural manifestations (**A**), exemplary red arrowheads, maximum standardized uptake value (SUVmax) up to 25.2). Additionally, the baseline staging revealed an intense focal (peri)muscular glucose metabolism in the lateral part of the right musculus gluteus minimus without any correlation on low-dose CT (**A**), green arrowhead, SUVmax 26.1). This finding was initially thought to be an additional extranodal manifestation of lymphoma. While all other nodal and extranodal manifestations of NSHL demonstrated complete metabolic remission after induction therapy, the finding remained with unchanged elevated glucose metabolism (**B**), green arrowhead, SUVmax 19.1). For further clarification, magnetic resonance imaging (MRI) was performed showing a circular, marginal contrast-enhancing lesion of approx. 2 cm diameter with central sparing and positive fascial tail sign (Figure 2A). A subsequent MRI-guided biopsy revealed an EBV-negative spindle cell lesion reactive with monoclonal antibodies to alpha-actin (1:700; clone 1A4; DAKO Agilent Technologies, Santa Clara, CA, United States) and classified as a solitary nodular fasciitis (Figure 2B,C), a benign soft tissue tumor, which is characterized by a fast-growing nature and high mitotic count [1,2]. While prevalence of nodular fasciitis in the pediatric population is relatively low, with only 10% of all reported cases being children, tumor location in the muscles of the lower extremities is rarely reported, with the most common tumor sites being described in the upper extremities or in the head and neck [3,4,5]. The observed [^18^F]FDG avidity of the nodular fasciitis lesion is presumably based on the increased glucose utilization due to the high proliferation rate. Clear differentiation of nodular fasciitis from viable extranodal manifestation of malignant lymphoma appears to be challenging on [^18^F]FDG PET/CT imaging [6,7,8,9]. In addition, there is a variety of other known benign diseases which could also mimic malign manifestations on [^18^F]FDG PET/CT [10,11,12,13,14].

Furthermore, it is known that lesions of nodular fasciitis tend to also show increased uptake of PSMA-targeted tracers, which may cause potential pitfalls for other tracers as well [15,16]. Magnetic resonance imaging seems to be a feasible complementary modality for further non-invasive differentiation [17,18]. This interesting image should draw attention to considering nodular fasciitis as a possible pitfall in the staging of malignant diseases on [^18^F]FDG PET/CT.

**Figure 2:**
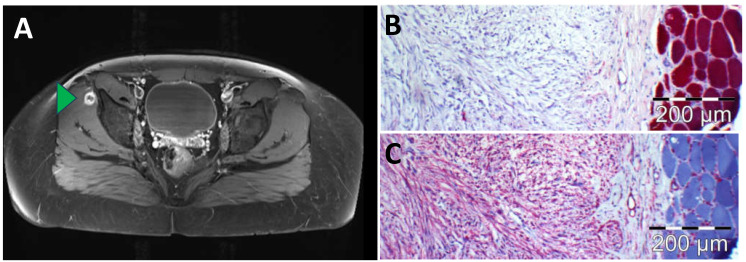
(**A**) Magnetic resonance imaging (MRI) with contrast-enhanced T1 TSE (Turbo Spin Echo) sequence and fat saturation showing a circular, marginal contrast-enhancing lesion with central sparing and positive fascial tail sign (green arrowhead); (**B**) immunohistochemistry revealed that lesional spindle cells hardly interact with anti-alpha gamma actin; (**C**) immunohistochemistry disclosed that lesional spindle cells strongly react with antibodies to alpha actin.

## Data Availability

The datasets used and analyzed in this paper are available from the corresponding author on reasonable request.
